# Diseases of the Fallopian Tubes

**Published:** 1885-08

**Authors:** 


### Diseases of the Fallopian Tubes.

Any cause which may produce endometritis may produce salpingitis by extension of the inflammation into the fallopian tubes. Gonorrhœa in the female is probably a very frequent cause of the disease. The sterility so universal among prostitutes is probably explained by the fact that gonorrhœa and septic endometritis, following abortions, are so common in this class.

Septic poisoning, following labor and abortions, especially the latter, is a frequent cause of this disease. By extension of the inflammation to the tubes, the fimbriated extremity may become closed, accompanied by a local peritonitis. Strictures of the tube may occur at various points, thus sacculating, as it were, the secretions of the mucous membrane. These may rupture into the peritoneal cavity, producing repeated attacks of local peritonitis. The character of the secretion, whether it be pus or semen, determines the accumulations as being pyo-salpinx or hydro-salpinx. Hydro-salpinx may be produced by other causes than venereal disease, but the fact is not satisfactorily explained.

The symptoms from which to make a diagnosis are variable. The most constant is a peculiar burning pain over the seat of the affected tube. Many have only a local sensitiveness, and a dull, heavy, dragging pain over the region of the ovaries, with backache and headache, so commonly supposed to be associated with displacement. Displacement is a very constant accompaniment of diseased tubes, and often produced thereby.

Sterility is the rule in salpingitis if both tubes are diseased, and they usually are. If one is not, pregnancy may occur, but abortion is liable to follow, because of the adhesions of the tube upon the diseased side.

The most probable reliable symptom is the frequent occurrence of local peritonitis, or pelvic congestion, without any other good cause for such attacks.

An acute attack is not readily diagnosticated. After rest in bed, and applications of pledgets of cotton, saturated with glycerine, to the cervix and vault of the vagina, two or three times a week, for several weeks, the inflammatory products will probably be so far absorbed as to render diagnosis possible by bimanuel palpation. Salpingitis is said to be more frequently associated with antero-lateral displacements, than with retro-displacements, the adhesions formed tending to draw the organ forward and laterally. A small percentage, however, accompany retroversion; when only one tube is diseased, it is usually the left.

As the disease progresses, more commonly the tube becomes distended by fluid, assuming small or enormous dimensions. When the accumulation of fluid is large, the tube is usually convoluted and constricted at one or more points, the outer end ordinarily being larger than the inner end.

The fluid may be clear, milky, thick, greenish pus, or thin pus. Microscopical examination of the clear fluid shows its chief characteristic to be ciliated epithelium. When the tube is largely distended with few adhesions, it is usually a hydro-salpinx; but when many adhesions exist, the fluid is generally purulent—a pyo-salpinx.

Salpingitis always renders the patient an invalid. A pyo-salpinx ordinarily causes an almost constant state of partial septicemia, with slight fever and rise of pulse. A hydro-salpinx may at any time become a pyo-salpinx; therefore, operative procedure in undoubted cases is indicated. If the patient objects to laparotomy and removal of the diseased appendages, tapping of the accumulation, if large, through the vagina, may be resorted to, perchance followed by complete recovery—perhaps not.

A trocar and canula are introduced, the trocar removed, a probe passed into the cavity, the canula removed, a dilator passed alongside the probe, the opening dilated and a soft rubber drainage tube introduced and retained by stitching it to the cervix. Then follows the irrigation of the cavity with antiseptic solutions, preferably the mercuric bichloride, till granulations fill the sac. This procedure is advised when only one cyst exists.

At any time, a patient with pyo-salpinx is in danger of rupture of the distended tube and death from general peritonitis.

A rupture followed by peritonitis should be operated upon, the tube removed and the peritoneum cleansed and drained.

Dr. Wylie believes that a careful study of the diseases of the fallopian tubes will account for most of the cases of l[illegible]al peritonitis, and most of the cases of retroversion, retroflexion and lateral flexions with adhesions, and that their proper treatment will show the uselessness and danger of attempting to use pessaries in these cases.

